# Emerging Applications of Electrochemical Impedance Spectroscopy in Tear Film Analysis

**DOI:** 10.3390/bios12100827

**Published:** 2022-10-05

**Authors:** Berin Ozdalgic, Munire Gul, Zihni Onur Uygun, Nazente Atçeken, Savas Tasoglu

**Affiliations:** 1 Department of Mechanical Engineering, Engineering Faculty, Koç University, Istanbul 34450, Türkiye; 2 Koç University Translational Medicine Research Center (KUTTAM), Koç University, Istanbul 34450, Türkiye; 3 Division of Optometry, School of Med Services & Techniques, Dogus University, Istanbul 34775, Türkiye; 4 Department of Biochemistry, Faculty of Medicine, Kafkas University, Kars 36100, Türkiye; 5 Boğaziçi Institute of Biomedical Engineering, Boğaziçi University, Istanbul 34684, Türkiye; 6 Koç University Arçelik Research Center for Creative Industries (KUAR), Koç University, Istanbul 34450, Türkiye

**Keywords:** biosensing, ocular diagnosis, systemic disease diagnosis, tear fluid, electrochemical impedance spectroscopy

## Abstract

Human tear film, with a flow rate of 1–3 µL/min, is a rich bodily fluid that transmits a variety of metabolites and hormones containing proteins, lipids and electrolytes that provide clues about ocular and systemic diseases. Analysis of disease biomarkers such as proteins, mRNA, enzymes and cytokines in the tear film, collected by noninvasive methods, can provide significant results for sustaining a predictive, preventive and personalized medicine regarding various diseases such as glaucoma, diabetic retinopathy, keratoconus, dry eye, cancer, Alzheimer’s disease, Parkinson’s disease and COVID-19. Electrochemical impedance spectroscopy (EIS) offers a powerful technique for analyzing these biomarkers. EIS detects electrical equivalent circuit parameters related to biorecognition of receptor–analyte interactions on the electrode surface. This method is advantageous as it performs a label-free detection and allows the detection of non-electroactive compounds that cannot be detected by direct electron transfer, such as hormones and some proteins. Here, we review the opportunities regarding the integration of EIS into tear fluid sampling approaches.

## 1. Introduction

Early diagnosis of systemic diseases, monitoring the response to a particular treatment, understanding the natural course of the disease and development/testing of point-of-care devices and new drugs [1,2] depend on pro re nata and target-specific measurements via noninvasive methods. In addition to physical examination, such diagnostic testing methods are vital in the interpretation of observed symptoms and in the early diagnosis of clinically asymptomatic conditions. These diagnostic testing methods can be broadly classified as laboratory medicine, anatomical pathology and medical imaging [3]. Of these, anatomical pathology and medical imaging involve specialized operators, invasive methods and high cost [3]. On the other hand, collecting biological fluid samples is an assertive and practical method that has been used for many years to discover specific biomarkers by proteomic and genomic studies and to utilize them in diagnoses based on normal ranges defined from samples of healthy individuals [4]. Although there are several body fluid sources for biomarker analysis, namely blood, serum, cerebrospinal fluid (CSF), urine, saliva, sweat, tears, etc., we cannot generalize them all as noninvasive or practical in terms of their extraction, collection or analysis methods. For instance, blood and CSF samples are collected by invasive methods. CSF extraction and biopsy require hospitalization and should be operated by an expert, moreover, these practices carry high risks and may not be possible to be applied to every patient [1,2]. Analyzing blood and urine samples requires costly devices and some preliminary steps [2]. Saliva and CSF can be contaminated by blood or microorganisms. In contrast, tear fluid is a significant source of biomarkers that is practically extracted without contamination and does not require pretreatment for the assay [1].

Tear fluid is a meaningful biomarker source and specific diseases showed distinguished proteomic patterns in tear fluid. Several studies to date have found that tears contain diverse diagnostic indicators of ocular diseases, namely glaucoma, dry eye disease, keratoconus, keratopathy, thyroid eye disease, diabetic retinopathy, etc., and systemic diseases, namely cancer, Parkinson’s disease, Alzheimer’s disease, COVID-19, etc. [1,4]. In particular, some studies showed that breast cancer patients have a distinguished proteomic pattern in tear samples than from control groups [5,6,7,8,9]. In non-contact lens wearers, β2-microglobulin levels together with PRP4, lysozyme C and cystatin may serve as a distinguishing biomarker for thyroid-associated orbitopathy (TAO) [10]. For Alzheimer’s disease, total tau has the potential to be a distinctive biomarker together with other disease markers determined by proteomic studies in tears [11,12,13]. For sustaining a preventive and personalized medicine based on tear film, various biosensors have been developed to detect tear biomarkers with high sensitivity [4]. There is an increasing number of studies focusing on developing biosensors for the detection of diabetic retinopathy, diabetes, whole-body hydration status and the levels of vitamins, glucose, cortisol and alcohol in tears collected by noninvasive methods [1,14]. Biosensors are devices that are mostly designed to quantitatively detect disease biomarkers in human samples, providing fast and accurate results. A biosensor is composed of a biorecognition receptor to interact with a target molecule and a physiochemical transducer such as electrodes. In these devices, a transducer detects the interaction between the target analyte and a biorecognition receptor, involving antibody/antigen, enzymes/ligands, nucleic acids/DNA, cellular structures/cells or biomimetic materials, and generates a measurable signal for the detection of the target biomarker in the sample fluid [15,16,17]. Various sensors have been developed to measure the interaction of disease biomarkers in body fluids with different types of biorecognition elements, but the portable or wearable ones are in the center of interest because they provide quick and convenient testing at the point of care. Portable biosensors can be classified by the transducing technique as piezoelectric, optical, colorimetric or electrochemical biosensors [18]. Among them, electrochemical impedance spectroscopy (EIS) is an effective and quantitative method that detects electrical equivalent circuit parameters related to biorecognition events on the electrode surface [19]. EIS is performed for the determination of the circuit model of biosensor surface. In biosensor technology, the elements are altered to the detection and surface modifications. In general, EIS detection recognizes the biosensor construction qualitatively and target molecule detection quantitatively. Therefore, the equivalent circuit model is crucial for accurate detections. The model is prepared by using the obtained Nyquist plot data, which generally have a sinusoidal and linear part. The sinusoidal part represents electron transfer resistance of the electrode surface (Rct) and redox probe solution resistance (Rs). There is also the electrical charge originated from the electrode surface layers as capacitance. The capacitance represents the electrical properties of the electrode, however, in different studies such as non-homogeneous surfaces, capacitance should be replaced to the constant phase element to overcome misfitting of EIS data to the equivalent circuit model. AC- and DC-based detections provide advantages as they perform a label-free detection and allow the detection of non-electroactive compounds that cannot be detected by direct electron transfer, such as hormones and some proteins. Known to electrochemists for over a century, electrochemical impedance spectroscopy (EIS) has become increasingly popular over the past decade as a label-free detection tool with many different types of biosensors based on biorecognition elements [18,20,21].

There are many studies claiming that the glucose level in tears is compatible with that in blood [22,23]. High-sensitivity glucose sensors using electrochemical interaction, fluorescence and reflectance spectroscopy-based methods have been developed for the determination of glucose in tears, in order to play a role in the early diagnosis of diabetes, a serious disease [14,24,25,26,27]. Uric acid level detection is performed by an amperometric electrochemical sensor depending on the electrooxidation of uric acid in tears. As in blood, uric acid levels in tears can provide significant indicators of hypertension, stroke, gout, Alzheimer’s disease or cardiovascular diseases [28]. Diabetic retinopathy, another serious disease, can damage optic nerves and cause vision loss. Optoelectronic and optoelectrokinetic bead-based immunosensors for lipocalin-1 detection [29,30] and Au-NP-doped diffusometric immunosensors for TNF-α detection have been developed to diagnose diabetic retinopathy in tears [31]. Apart from that, there have been many tear-based biosensors developed to provide information about the body’s hydration status, alcohol intake or cortisol levels [14,32,33]. Some of these are designed as wearable devices and their in vivo studies have provided promising results [18,22,27,29,30,34,35]. Studies in which EIS-based wearable tear-detection technologies have been developed and tested are limited and have not yet been tested on living systems.

**Figure 1:**
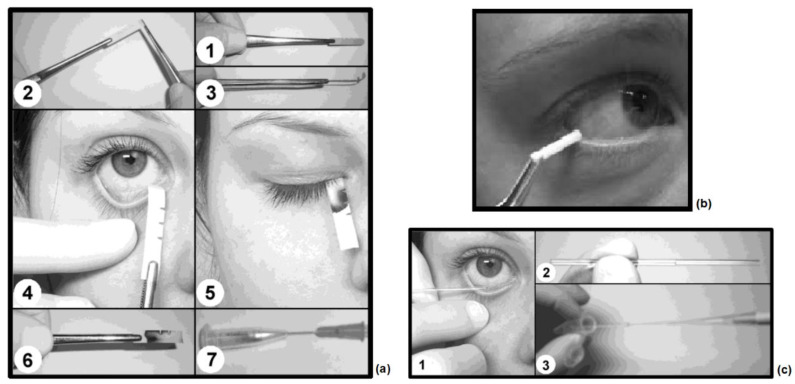
Tear fluid collection methods. (**a**) Sampling with Schirmer’s test: The filter paper is folded over at right angles (1–3) and inserted into the lower conjunctival sac above the punctum lacrimale using a forceps (4). During sampling, eyes are kept closed in neutral position (5). For removal with forceps, eyes are opened, the lower lid is pulled down and the strip is removed by pulling it upward and out of the eye (6); after an average time of 5 min, the strip is completely moistened. Test strip transferred into a collection tube (7) (Reprinted with permission from Ref. [36]. Copyright 2012 Elsevier). (**b**) Sampling with surgical sponge rod: While the rod tip is resting in the lower tear meniscus, cellulose rod is placed close to the lower lid by holding a forceps (Reprinted with permission from Ref. [37]. Copyright 2008 John Wiley and Sons). (**c**) Sampling with capillary tube: To obtain tear fluid, the lower lid is pulled downward and while holding the tube laterally from the inferior temporal tear meniscus toward the outer canthus of the eye (1), a capillary tube extracts tear fluid from lower cul-de-sac (2) and extracted samples are transferred into a collection tube (3) (Reprinted with permission from Ref. [36]. Copyright 2012 Elsevier).

Tears can be collected simply and quickly with conventional methods, but these methods should be applied carefully to not damage the ocular surface [1]. Collection methods may affect the composition of the tear samples due to the presence of sensory nerves on the ocular surface [36,37]. Schirmer’s strip and surgical sponge methods rely on the collection of tears by absorption while in contact with the conjunctiva. For this reason, these methods cause excessive secretion of reflex tears by stimulating the sensory nerves on the ocular surface [1]. It is also critical to pay attention to not damage the ocular surface while the absorbent is in contact with the conjunctiva. On the other hand, the microcapillary tube method is a less invasive approach and the application does not cause excessive reflex tear secretion as the tube has no contact to the cornea, conjunctiva and lower eyelid [1,38]. In this method, a glass microcapillary tube extracts tear fluid while holding the tube laterally from the inferior temporal tear meniscus toward the outer canthus of the eye [1,36,38]. The amount of sample collected by this method is less in proportion to the amount of reflex fluid released, but the concentration of samples collected will be higher than the absorption methods. The application procedures of all three methods are shown in Figure 1. Samples collected by traditional methods are transferred into a collection tube. In Schirmer’s test and surgical sponge methods, a small hole is made in the tip of this collection tube and then this tube is inserted into a larger Eppendorf tube. Finally, this combination is spun in a centrifuge [37].

We mentioned above that anatomical pathology and medical imaging as well as laboratory medicine may require qualified labor and equipment. Although tear fluid can be extracted more quickly and practically, collecting body fluids with traditional methods requires additional procedures and care. In this review, we explain the advantages of methods for collecting and analyzing tear fluid as a biomarker source, in which the molecules in the blood can easily diffuse. Furthermore, we discuss the advantages of electrochemical impedance spectroscopy (EIS) in tear fluid analysis over other methods through the potential future aspect of EIS based point-of-care and wearable devices.

## 2. Physiology of Tear Film

The ocular surface is covered by the tear film, and this layer keeps the corneal and the conjunctival epithelia safe from the outer conditions. The tear film is composed of three layers: the inner mucous layer, the middle aqueous layer and the outer lipid layer. While the inner mucous layer secretes from the conjunctival goblet cells, the lacrimal gland produces the middle aqueous layer and the outer lipid layer is released from the meibomian glands. The lacrimal glands are placed in the upper lateral region of each orbit and have an almond-like shape. These glands, which are made of connective tissue, are composed of lobules containing many acini. Serous or mucous acinar cells, which secrete fluid into the central lumen by contraction of myoepithelial cells (basket cells), are composed of many acini that form the spatial organization of these cells. The secretion from the lumen flows from the ocular surface, which consists of many channels, towards the puncta lacrimalia. After these secretions are collected in a lacrimal sac, they are poured into the nose through the lacrimal duct, formed by duct epithelial cells [39]. The lacrimal glands, lacrimal canaliculi, lacrimal sac, and nasolacrimal duct form the lacrimal system. The lacrimal gland is a serous and compound tubuloalveolar gland. The secretory acini in this gland are surrounded by myoepithelial cells. The gland is placed outside the conjunctival sac. The lacrimal fluid secretion (tears) drains into the conjunctival sac via 6 to 12 secretory ducts. The structure of the tear, which is poured into the conjunctival sac through the secretory ducts, is mostly composed of water. This liquid compound mainly contains antibacterial lysosomes [40]. The tear film that covers the cornea and conjunctiva layers consists of tear fluid. The tear film has an inner mucin mucous layer, a middle aqueous layer and an outer lipid layer. The water-retaining and viscous nature of the mucous layer may enhance the drench properties and stability of the whole tear film. Many different proteins are placed in the aqueous layer. These kinds of proteins are interested in wound healing and inflammatory processes, as well as corneal protection from various pathogens. Tear fluid is important for the ocular surface to keep it in proper function and health. Some of the tasks of the tear fluid are humidifying the ocular surface, sweeping contaminants out of the eye, guarding the ocular surface against pathogens, lubricating the lid–cornea interface when blinking and sleeping and nourishing corneal epithelial cells. Moreover, this fluid helps to improve optical properties by modifying the refractive index of the cornea [41]. Tear fluids contain mucins, glycoproteins, unglycosylated proteins, peptides and lipids as exclusive body fluids [42]. Lysozyme, lactoferrin, secretory immunoglobulin A, lipocalin and lipophilin are the major tear proteins.

### Tear Fluids as Biomarker

Tear fluid can be considered as an ideal biomarker source because the body is exposed to different pathophysiological conditions during disease periods and the tear content changes accordingly [43]. In parallel, it has been reported that the protein components of tears are altered in some ocular diseases or nonsystemic and systemic diseases. [8,44,45,46]. According to proteome analysis of tears, this fluid has approximately 500 proteins; on the other hand, plasma may contain up to 10,000 proteins [47,48]. Furthermore, since sampling is less invasive, more accessible and less complex than other body fluids such as serum or plasma, many researchers have focused on the changing proteomic, lipidomic and metabolomic compositions of tear film during the disease periods. Determining the compositional changes of tear fluid profiles is significant to identify pathways of some disease’s progression. Correspondingly, meeting the personalized therapy for individuals may be more predictable [4].

Many novel protein-based biomarkers have been correlated to specific ocular diseases, namely dry eye syndrome, trachoma, glaucoma, keratoconus, allergic conjunctivitis, allergy with corneal lesions, blepharitis, conjunctivochalasis, mycotic keratitis, pterygia, thyroid-associated orbitopathy (TAO), etc., and systemic disorders, namely diabetes mellitus, cancer, systemic or multiple sclerosis, cystic fibrosis, Parkinson’s disease, sclerosis, etc. [43,49,50]. To answer clinical questions, many methods have been used to identify quantitative changes in protein expression in a biological system. Proteomic approaches are the most frequently used method to identify and precisely quantify thousands of proteins from simpler or complex samples extracted from the body. There are many studies on detection of tear-based protein biomarkers with proteomic techniques. With proteomic techniques, a large number of peptide/protein separations as well as the identification of these proteins can be performed in a very short time. Especially with the help of these technologies, many tear-based protein biomarkers can be used for diagnostic purposes in various cancer diseases [43]. Proteomic techniques are divided into gel-based and non-gel-based mass spectrometry (MS). Surface-enhanced laser desorption/ionization time-of-flight (SELDI-TOF) and matrix-assisted laser desorption/ionization time-of-flight (MALDI-TOF), high-pressure liquid chromatography (HPLC) and two-dimensional electrophoresis (2-DE) are the main utilized techniques [51,52]. Cytokines, growth factors and mucins are the best-known tear biomarkers. In multiplexed cytokine analysis, the cytometric bead-based assay (CBA) system, which is a combination of flow cytometry with enzyme-linked immunosorbent assay (ELISA) provides ultra-highly sensitive results [53]. With these techniques, thousands of protein analysis can be performed by tear fluid with a volume of 1–5 microliters. In addition, tear fluid is a unique source of protein compared to other body fluids. Namely, basal tear film has 7 mg/mL protein content, while urine contains only 0.049 mg/mL protein [54]. Accordingly, a summary of the tear-based biomarkers for the diagnosis of specific diseases, as well as biomarkers for evaluating the efficacy of treatments for chronic glaucoma patients and regular contact lens wearers are given together with the utilized techniques including conventional and EIS in tear detection in Table 1**.**

## 3. Emerging Applications of Tear-Based Electrochemical Detection

Although the use of tear fluids as a diagnostic tool has advantages over other body fluids such as blood, CSF, sweat and saliva, there are some difficulties in collecting tear fluids under the right conditions and without damaging the eye. In addition, it is critical to analyze target biomarkers with sensitive and reliable techniques that can perform repetitive studies despite the small number of samples.

Biosensors are analytical devices that measure the binding to specific target analytes by using appropriate biorecognition receptors, and this interaction forms measurable electrochemical, optical, thermal or mass-based signals. Recent studies on biomedical applications have focused on electroanalytic detection techniques as they potentially offer quick and relatively low-cost monitoring. Electrochemical sensors basically consist of two main components. One of these components is a biorecognition receptor system such as enzymes, DNAs, proteins and antibodies that recognize analyte; and the other is a physicochemical transducer that easily detects and displays interactions through electrical-based components and converts them into electrical signals. Electrochemical sensors can be divided into main classes such as conductometric, voltametric and potentiometric according to electroanalytical technique [79,80]. Electrochemical detectors are well-established and powerful tools for obtaining real-time information for process control through in situ measurements of chemical composition. In addition, they are simpler in terms of electronic equipment used for installation, operation and data collection compared to spectrometric (FTIR, UV-VIS), mass spectrometric (MS) and chromatographic techniques (GC, HPLC) [81]. Electrochemical biosensors in particular have a significant advantage over optical biosensors in that they offer label-free measurement and allow for measurement within a heterogeneous sample [82]. For this reason, they offer a lower cost and convenient measurement opportunity compared to other methods. Another advantage of biosensor technology is the possibility of working with low sample volume. This is the case of measuring without providing a pretreatment to the sample. In this way, more efficient and accurate analysis can be performed. The analyses to be performed on the tears allow for a painless analysis of the patient, especially with the noninvasive sampling of the sample. In this way, it emerges as a preferred method of sampling these days, where personalized medicine gains importance. Tears are important because they contain biomarkers that can provide information about metabolic diseases as well as providing information about the general eye area [83]. These biomarkers can be protein-based or various metabolites. For this reason, measurements can be made only by sample extracting, which facilitates the analysis. For example, in the case of occlusal allergy, cytokines (interleukins) and immunoglobulins can rise in eye infections, and bacteria/parasites can be found in tears [4]. Considering these biomolecules, electrochemical systems based on electroactive molecule measurement cannot be used to measure biomarkers other than metabolites such as glucose, because bacteria measurements in particular cannot be performed electroactively. Therefore, label-free electrochemical measurement methods should be preferred [84,85]. Here, electrochemical impedance spectroscopy takes a step forward in terms of sensitivity and selectivity. Apart from this, with EIS it is possible to measure biomolecules and characterize the biosensor system at the same time. This is not possible with other methods, and the measurement of electroactive molecules is open to interference. The need for a secondary molecule for some measurement is a significant disadvantage that can be overcome by using the EIS method. Since EIS can also be used for the characterization of tear film layers, it constitutes an important and strategic measurement method among other electrochemical measurement methods [84,85,86]. It is critical to analyze this sample type, which has a significant place in the determination of systemic and ocular diseases yet obtained in low quantities, with a powerful system such as EIS, which is highly sensitive and does not require preprocessing.

The use of EC sensors and devices is highly involved in the biomedical as well as the agriculture, environmental sensing and defense industries [82]. In particular, the electrochemical impedance spectroscopy technique (EIS) can be adapted for molecular coupling-based applications and be used in plasmonic-based applications [87]. The use of electrochemical impedance spectroscopy, especially in biosensor and sensor technology, also enables surface characterization in the experimental data and development stages. In the methods used for the measurement of other electroactive molecules, the electroactive reduction/oxidation signals of the measured biomolecule or label are obtained. While this only gives electrochemical data about the molecule being measured and its concentration, measurements with EIS are used to reveal the electrochemical characteristics of the system being measured, as well as providing a label-free measurement [84]. Here, the surface resistance of the material, that is, the electron transfer resistance, measures the resistance of the solution being measured. On the other hand, as a result of the interaction on the electrode, that is, the biosensor technology used in the measurement, it can simultaneously measure the mass transport transfer resistance (Warburg impedance) moving to the surface and the double layer capacitance of the electrode layers [85]. In this way, the electrode–electrolyte interface can be examined in the best way. The first and main purpose of the measurement in impedance spectroscopy is to extract the surface circuit model of the biosensor. Since the data obtained in the measurement are a geometrical feature of the electrode, it makes it easy to understand and optimize the measurement, even if a new-generation transducer is used. Although complex mathematical transformations are used to explain the elements in the circuit model, calculations can be performed easily in biosensor applications [86]. Calculation of the data obtained by impedance measurement by sitting on the circuit model, that is, “fitting”, is possible by knowing the theoretical properties about the surface beforehand. At this point, it is important to define the obtained data with a suitable circuit model, because when examining the data obtained in the form of a Nyquist plot, the circuit model is formed according to this curve. The characteristic of this curve is completely related to the electrode surface. In this way, impedance spectroscopy is an extremely effective and convenient method, especially for the determination of the characteristics of the studies to be carried out, apart from the electrochemical measurement.

**Figure 2:**
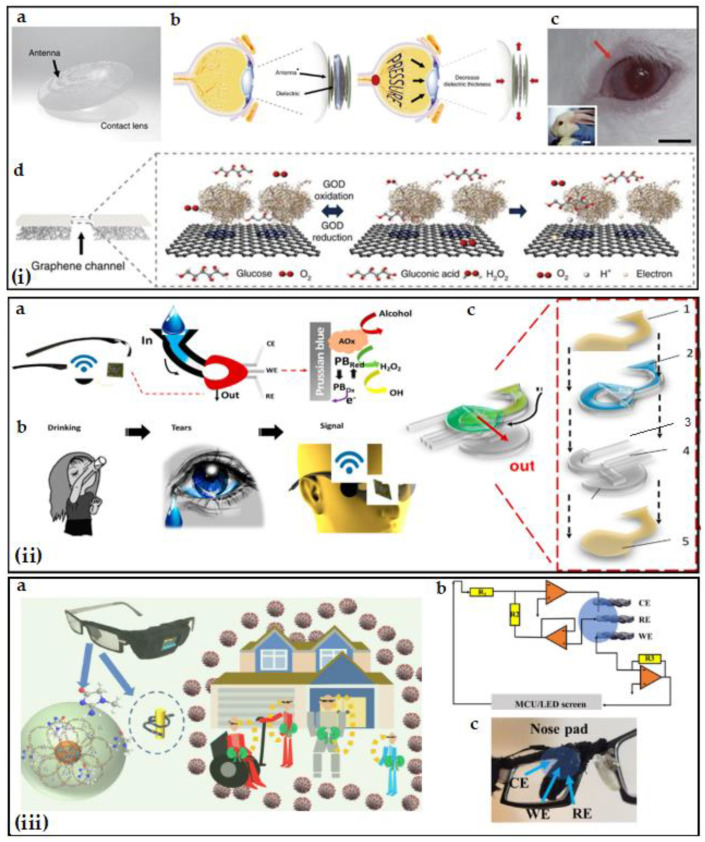
Real-time tear detection via wearable electrochemical sensors. (**i**) Smart contact lens for glucose and IOP detection. (**a**) Schematic illustration of the smart contact lens with IOP and glucose sensing. (**b**) Schematic illustration of working mechanism for IOP detection. (**c**) Photograph of the sensor integrated onto the eye of a live rabbit. Black and white scale bars, 1 cm and 5 cm, respectively. (**d**) Schematic illustration of working principle of glucose detection (Reprinted from Ref. [34]). (**ii**) Lab-on-eyeglasses for alcohol detection. (**a**) Schematic illustration of the lab-on-eyeglasses and working mechanism for alcohol detection via enzymatic measurement. (**b**) Schematic illustration of the procedure. (**c**) Structure of the fluidic device: (1) top polycarbonate membrane, (2) double-adhesive paper, (3) paper outlet, (4) electrochemical sensor and (5) bottom polycarbonate membrane (Reprinted with permission from Ref. [14]. Copyright 2019 Elsevier; Reprinted with permission from Ref. [91]. Copyright 2022 American Chemical Society). (**iii**) Lab-on-eyeglasses for creatinine detection. (**iii**) (**a**) Representative image of lab-on-eyeglasses with disposable hybrid textile electrodes selective for tear creatinine to monitor kidney diseases during daily life. (**b**) Circuit design of a textile-based integrated electrochemical sensor microcontroller with real-time monitoring. (**c**) Photograph of the three electrodes integrated into the nasal pad of the lab-on-eyeglass (Reprinted with permission from Ref. [63]. Copyright 2021 American Chemical Society).

Impedimetric sensors, which can be built with disposable screen-printed electrodes (SPEs), can completely eliminate the lengthy processes of immunoassay/ELISA. SPEs can be produced depending on the target molecule to be studied [14,87]. These electrodes, which showed superior repeatability of ~5% in blood glucose level tests, can be studied in small volumes and do not require surface pretreatment. In particular, EIS is an ultrasensitive technique with detection limits in the picomolar range [87]. Rohrbach et al. developed a sensor for lysozyme detection, which is a biomarker for blepharitis, dry eye syndrome, glaucoma and Alzheimer’s diseases, by using screen-printed electrodes [88]. In a recent study, amyloid concentrations were detected by cyclic voltammetry (CV) and electrochemical impedance spectroscopy (EIS) [61]. In another study, for detection, a handheld electrochemical impedance immunosensor and a disposable test strip for tear collection were developed to determine cortisol quantification within ~90 s and limit detection of 59.76 nM with a relative standard deviation of 10%. In this study, electrochemical measurements were based on cyclic voltammetry, square wave voltammetry and amperometric i-t [62]. With the acceleration of point-of-care studies, wearable technologies have started being used frequently in medical applications. Wearable technologies developed through electrochemical sensors can be used as an alternative to proteomic and conventional systems. Kalasin et al. [63] developed a lab-on-eyeglasses for point-of-care (POC) kidney monitoring by measuring tear creatinine selectively and precisely (Figure 2). This device measures charge-transfer resistances with a textile-based electrode by EIS to detect creatinine in tears. The advantages of this device are that it is wearable and can perform both tear collection and detection in one step with 95.1% selectivity and 96.2% sensitivity [63]. In addition to biomarker analysis, electrical impedance spectroscopy is an alternative measurement technique for osmolarity measurement. Testing tear film osmolarity can be an effective diagnostic tool in the diagnosis of dry eye disease. Measurement results obtained from keratoconjunctivitis sicca patients exhibited significantly higher tear film osmolarity than healthy subjects [89]. In another study, the EIS technique was used in a study comparing reflex and basal tear osmolarity values. The results showed no significant difference between basal and reflex tear osmolarity. This result may indicate that the tear-collection techniques that we explained in detail above do not affect the sample content and results [90].

In tear-based wearable technologies, electrochemical hardware is integrated into interfaces such as contact lenses, eyeglasses, nasal pads and spring-like sensors. For example, by means of polydimethylsiloxane (PDMS) covered with a hydrogen peroxide-permeable poly membrane, the glucose level in tears can be measured amperometrically with a glucose oxidase (GOx)-based glucose sensor [92]. Kim et al. [34] aimed to measure glaucoma and diabetes simultaneously with hybrid electrodes that they integrated into a contact. In this study, this multifunctional lens independently measured glucose levels with a GOD-pyrene functionalized graphene and intraocular pressure with a silicone elastomer (ecoflex) sandwiched by graphene-AgNW-based inductive spirals. A reader antenna was placed with an RLC passive circuit for wireless sensing [34]. Figure 2 shows the schematic Illustration of the transparent device and its position on a rabbit eye. The glucose level in tears was determined by the detection of the oxidation of hydrogen peroxide, which is a widely used technique, and the resonance frequency of the mechanical change on the device caused by intraocular pressure. In another study, Kagie et al. [93] developed a design that allows for minimally invasive amperometric monitoring of biomarkers in tear fluid. In this design, a miniature, flexible, thick-film electrochemical biosensor flow detector is placed in the tear duct. In general, these strips have mostly been utilized for keratoconjunctivitis sicca and, transcutaneous monitoring of oxygen and glucose levels.

Although strip-based ocular sensors offer a minimally invasive alternative to various techniques such as capillary microelectrophoresis and low-volume sampling [93,94], long-term storage of these sensors is difficult. Some plastic-based materials cause eye irritation and their use has not been very common as they cause reflex tears depending on the application. As another wearable system, contact lens-based sensors have advantages such as continuous sampling and ease of use, as well as oxygen permeability and continuous monitoring [50]. Based on this technology, a study was reported in which a GOx-based amperometric glucose sensor equipped with an in-built wireless readout chip was developed by [94]. Accordingly, the same working group developed a contact lens with a dual-sensor feature with activated and deactivated GOx to reduce invasive effects to improve the current system [95]. In another tear-based wearable system, it was reported that a high-sensitivity hydrogel field effect transistor (FET) was developed as a glucose sensor electrode, which has the potential to significantly reduce signal noise caused by nonspecific adsorption [96].

In another innovative wearable technology, a microfluidic electrochemical detector is placed on the nose bridge pad, providing the opportunity to eliminate the risks such as infection or visual impairment that may occur due to the use of contact lens systems. In this noninvasive system, stimulated tear fluids can be directly collected and analyzed [14]. A wireless fluidic device consisted of a screen-printed three-electrode system integrated onto the eyeglasses’ nose bridge. Figure 2(ii) shows the schematic illustration of the nose pad device. Real-time alcohol level in tears was determined by alcohol-oxidase (EtOx) detection. Some examples of studies for the electrochemical detection of tear-based biosensors are listed in Table 2.

## 4. Conclusions and Future Perspective

Although tears can be collected simply and quickly with conventional methods, they should be applied carefully to not damage the ocular surface. Additionally, the collection method may affect the composition of the tear samples due to the excessive secretion of reflex tears by stimulation of sensory nerves during the procedure. For this reason, it is extremely critical to integrate detection systems into wearable devices that do not disturb the user in order to determine the diagnostic ranges of the biomarkers in the tear and to utilize the tear as an easy, effective and safe diagnostic tool.

The diversity of biomarkers for various ocular and systemic diseases contained in the tear fluid put it forth as a promising diagnostic tool, but the identification of distinctive biomarkers and validation of diagnostic ranges are issues that need to be studied for its use as a diagnostic tool. In clinical assessment, variability, validity and reliability are major concerns in biomarker analysis. Intraindividual variability may result from the daily conditions of individuals, the substances they are exposed to and the way these substances are metabolized. In addition, intraindividual or intraobserver variability may be due to the application procedure or laboratory errors. For this reason, epidemiological studies should be followed for biomarkers and group variables should be determined before it is used in diagnosis. In addition, it is expected that the results obtained will be in correlation with the blood values used as a diagnostic source in diagnosis. Sensitivity is another important group variable [2]. Sensors used in tear analysis, and especially EIS-based emerging technologies that have developed in recent years, have shown high sensitivity in biomarker detection, yet proving the repeatability of obtained results is a challenge that needs to be studied in the future. The scientific question and costs are always concerned in selecting biomarkers and the source of the biomarker for research. After the research budgets, the size and the cost of clinical trials followed by the equipment and per capita costs in practice are critical for decision making.

Point-of-care devices sensing with EIS-based detectors, which are in the early stages of their development, have many advantages, such as being a label-free technique with their capability to measure the binding between the receptor and the target molecule for electroactive and non-electroactive compounds. Electrochemical impedance spectroscopy (EIS) for tear detection offers a powerful analytical technique for analyzing various biomarkers in tears; however, analysis based on impedance measurement needs further insight to prove the accuracy and repeatability by laboratory and clinical trials. Wireless powering and data transfer is a major technological challenge that a wearable device must provide. However, the advantage of wireless operation comes with the disadvantage of integration and cumbersomeness of excess electromechanical equipment. In these technologies, miniature electrochemical sensors used with various materials (for example, paper, DNA and nanomaterials) play a critical role in analytical fields due to their high sensitivity, portability, ease of use and short analysis time [99]. Therefore, studies on miniaturization, simplified sensor configuration and hierarchical architecture should be developed. In addition, the wearability of a smart contact lens with sensing ability eliminates the need for a laboratory and experts, due to the fact that this device is in contact with the ocular surface. This physical contact with the cornea requires additional care in the design of the device. In this sense, the materials to be used should be biocompatible, transparent and flexible, and the device should not overheat, should not contain toxic substances and should not disrupt the daily life of the person wearing the sensor by impairing the vision or ocular homeostasis. Since the cornea is an avascular tissue, proteins, electrolytes and water are supplied to these tissues only by tear film. For this reason, the high oxygen permeability of the device, which will be in contact with the cornea, is a critical feature for it to be worn for a long time. Most of the tear analyte sensors developed to date are still in product development or clinical trial stages and have not yet been commercialized. The aforementioned challenges in repeatability and accuracy for biomarkers and tears, the development of wearable devices and their biocompatibility are challenges that must be overcome for clinical studies to be successful.

## Figures and Tables

**Table 1:** Tear-based biomarkers and analysis techniques used in the diagnosis of specific diseases.

	Disease	Tear-Based Biomarker	Detection Technique/Platform	Sampling Technique	Reference
Systemic Diseases	COVID-19	IgA antibody; IL-6 cytokine	ELISA	Schirmer strip	[55]
	Control group	Protein and urea	DCDRS	Capillary tube	[56]
	Breast, lung; colon, ovary; prostate cancer	Lacryglobin	2DGE	Schirmer strip	[8]
	Breast cancer	Cystatin-SA, 5-AMP-activated protein kinase subunit gamma-3, triosephosphate isomerase, microtubule-associated tumor suppressor 1, transferrin receptor protein 1, putative lipocalin 1-like protein 1, DNA damage binding protein 1; protein S100-A9, GTP-binding protein Di-Ras2 miR-21 and miR-200c	MALDITOF-TOF; SELDI-TOF-MS; qRT-PCR	Schirmer strip	[5,6,7]
	Eye tumor cancer	Cystatins C and lactoferrin	ELISA	Capillary tube	[57]
	Clinically isolated syndrome as a systemic disorder	Oligoclonal bands	IEF	Schirmer strip	[58]
	Diabetes	LCN-1, HSP27, and B2M	2DGE	Preweighed polyester wick	[59]
	Parkinson’s disease	Catecholamine proteins from S100 superfamily, peroxiredoxin-6, annexin-X5, glutathione-Stransferase-A1; apolipoprotein superfamily—ApoD, ApoA4 and ApoA1, TNF-α, α-synuclein	HPLC-ED	Schirmer strip	[60]
	Alzheimer’s disease	Dermcidin, total tau, amyloid β 42, microRNA 200b-5p, lysozyme-C, lactotransferrin, prolactin, lipocalin-1, lacritin; β-Amyloid	LC-MS; EIS; multiplex immunoassay	Capillary tube; Schirmer strip	[11,12,13,61]
	Cortisol detection	Cortisol	EIS	Capillary tube	[62]
	Overall renal health	Glomerular filtration rate (GFR)	EIS	Cotton swab	[63]
Ocular Diseases	Control group for open-eye	Four phosphorylation sites for tear lipocalin	LC-MS	Capillary tube	[64]
	Keratoconus (KC)	Cystatin-S, cystatin-SN, cystatin-SA, lipocalin-1, Ig-k chain C, Ig J chain, lipophilin C, lipophilin A, phospholipase A2, serum albumin	2DGE; MALDI-TOF	Merocel sponge	[65]
	Conjunctivochalasis (CCH)	S100 family (A8, A9, A4), guanosine triphosphatebinding protein 2, L-lactate dehydrogenase A-like 6B, fatty acid-binding protein, keratin type I cytoskeletal 10, glutathione S-transferase P, peroxiredoxin-1, peroxiredoxin-5, cullin-4Bþ glyceraldehyde 3-phosphate dehydrogenase	2DGE; MALDI-TOF	Merocel sponge	[66]
	Allergic conjunctivitis	Serum albumin, Ig gamma-2, leukocyte elastase inhibitor	SDS-PAGE	Schirmer strip	[43]
	Allergy with corneal lesions	Human a-defensin	SELDI; ELISA verification	Capillary tube	[67]
	Blepharitis	Serum albumin, α1antitrypsin, lacritin, lysozyme, Ig-kappa chain V III, PIP, cystatin-SA III, pyruvate kinase	2DGE; Western blot verification	Preweighed polyester wick	[68]
	Pterygia	a-Defensins, S100 A8, A9	SELDI	Capillary tube	[69]
	Dry eye syndrome	Enolase, - 1-acid glycoprotein1, Calgranulin A, Calgranulin B, Calgizzarin, Prolactin Inducible protein (PIP), lipocalin-1, lactoferrin and Lyzozyme, Enolase	iTRAQ	Capillary tube	[69]
	Contact lens-related dry eye syndrome	β-2 microglobulin, PRP4, lacritin, secretoglobin 1D1, secretoglobin 2A2, serum albumin, glycoprotein 340, PIP	SDS-PAGE; 2D-DIGE; nano-LC–MS/MS	Capillary tube	[70]
	Non-Sjogren’s syndrome dry eye	Lipocalin-1, prolactin-inducible protein, and lysozyme PRP3, proline-rich protein4, nasopharyngeal carcinoma associated proline-rich protein4, a-1-antitrypsin, calgranulin A	iTRAQ; SELDI	Schirmer strip	[71,72]
	Sjogren’s syndrome dry eye	Proline-rich protein4	2DGE	Schirmer strip	[73]
	Mycotic keratitis	Glutaredoxin-related protein, PIP, serum albumin, cystatin S, cystatin-SN, cystatin, lipocalin	2DGE	Capillary tube	[74]
	Thyroid eye disease	Proteins: IL-10, IL-12-p70, IL-13, IL-1β, IL-2, IL-4, IL-6, IL-8 and IL-17A; TNF-α, RANTES; PAI-1, calcium-binding proteins, prolactin-induced protein, zinc-alpha-2 glycoprotein 1 Nonprotein molecules: 8-OHdG; malondialdehyde	Multiplex bead array; ELISA; Plex panel	Schirmer strip	[1] and references cited in there.
	Thyroid-associated orbitopathy (TAO)	PRP4, β2-microglobulin, lysozyme C, cystatin	SELDI; MALDI	Schirmer strip	[10]
	Diabetic retinopathy (DR)	Lactotransferrin, lacritin, Ig lambda chain C region, lipocalin 1, mammaglobin B and lipophilin A, lysozyme C Glycated; TNF-α and glycoprotein VEGF	ELISA; nanoHPLC coupled ESI; 1D- and 2D-SDS gels	Capillary tube; Schirmer strip	[1,75,76]
	Glaucoma	Proteins: Endothelin-1; kallikrein and ACE activity; lysozyme C, protein S100, lipocalin-1 and prolactin; cystatin-S; collagen type-IX and HNK-I epitopes, MMP-9, connective tissue growth factor Nonprotein molecules: Lysophospholipids and acetylcarnitine	LC-MS; MALDI-TOF; SDS-PAGE	Schirmer strip	[1] and references cited in there.
Ocular Treatment	Contact lens usage (rigid gas-permeable and soft contact lenses)	S100 A8, lysozyme, cystatin SELDI	Antibody microarrays	Schirmer strip	[77]
	Chronic glaucoma	S100-A8, S100-A9, mammaglobin B, and 14-3-3 z/d	iTRAQ	Schirmer strip	[78]

In the table, ELISA: enzyme-linked immunosorbent assay; DCDRS: drop coating deposition Raman spectroscopy; 2DGE: two-dimensional gel electrophoresis; MALDI: matrix-assisted laser desorption/ionization; SELDI: surface-enhanced laser desorption/ionization; TOF: time-of-flight; qRT-PCR: quantitative reverse-transcription polymerase reaction; IEF: isoelectric focusing; HPLC-ED: high-performance liquid chromatography with electrochemical detection; LC-MS: liquid chromatography-MS; EIS: electrochemical impedance spectroscopy; SDS-PAGE: sodium dodecyl sulfate polyacrylamide gel electrophoresis; iTRAQ: isobaric tags for relative and absolute quantitation; DIGE; differential gel electrophoresis; nano-LC-MS/MS: nano-liquid chromatography tandem mass spectrometry.

**Table 2:** Emerging applications of tear-based electrochemical detection platforms and target biomarkers.

Electrochemical Detection Platform	Target Biomarker	Monitoring Sensor	Reference
Strip-based sensor	Glucose	Glucose oxidase (GOx)	[92,93]
Smart contact lens	Glucose	Glucose oxidase (GOx)	[97]
Smart contact lens	Glucose	Dual GOx-based	[94]
Smart contact lens	Lactate	Lactate oxidase (LOx)	[98]
Smart contact lens	Glucose	Field effect transistor (FET)	[96]
Smart contact lens	Glucose, ocular pressure	Multifunctional electrical response elements	[34]
Spring-like sensor	Glucose	Polysaccharide-coated device (NovioSense Glucose Sensor)	[22]
Eyeglasses	Alcohol, glucose, vitamins	Alcohol-oxidase (AOx) biosensing fluidic system	[14]
Eyeglasses	Creatinine	Glomerular filtration rate	[63]

## Data Availability

Not applicable.
